# Deep Learning Architecture Reduction for fMRI Data

**DOI:** 10.3390/brainsci12020235

**Published:** 2022-02-08

**Authors:** Ruben Alvarez-Gonzalez, Andres Mendez-Vazquez

**Affiliations:** Department of Computer Science, Cinvestav Guadalajara, Zapopan 45015, Mexico; rodolfo.alvarez@cinvestav.mx

**Keywords:** CNN, machine learning, deep learning, computer vision, transfer learning

## Abstract

In recent years, deep learning models have demonstrated an inherently better ability to tackle non-linear classification tasks, due to advances in deep learning architectures. However, much remains to be achieved, especially in designing deep convolutional neural network (CNN) configurations. The number of hyper-parameters that need to be optimized to achieve accuracy in classification problems increases with every layer used, and the selection of kernels in each CNN layer has an impact on the overall CNN performance in the training stage, as well as in the classification process. When a popular classifier fails to perform acceptably in practical applications, it may be due to deficiencies in the algorithm and data processing. Thus, understanding the feature extraction process provides insights to help optimize pre-trained architectures, better generalize the models, and obtain the context of each layer’s features. In this work, we aim to improve feature extraction through the use of a texture amortization map (TAM). An algorithm was developed to obtain characteristics from the filters amortizing the filter’s effect depending on the texture of the neighboring pixels. From the initial algorithm, a novel geometric classification score (GCS) was developed, in order to obtain a measure that indicates the effect of one class on another in a classification problem, in terms of the complexity of the learnability in every layer of the deep learning architecture. For this, we assume that all the data transformations in the inner layers still belong to a Euclidean space. In this scenario, we can evaluate which layers provide the best transformations in a CNN, allowing us to reduce the weights of the deep learning architecture using the geometric hypothesis.

## 1. Introduction

Two recent neuroimaging studies [1,2] have decoded the structure and semantic content of static visual images from human brain activity. Considerable interest has developed in decoding stimuli or mental states from brain activity measured by functional magnetic resonance imaging (fMRI). The significant advantage of fMRI is that it does not use radiation, as is the case with X-rays, computed tomography (CT), and positron emission tomography (PET) scans. If performed correctly, fMRI poses virtually no risks. It can be used to safely, non-invasively, and effectively evaluate brain function [3]. fMRI is easy to use, and the produced images have very high resolution (as detailed as 1 millimeter). Compared to the traditional questionnaire methods of psychological evaluation, fMRI is much more objective.

Technological advances have led to significant evolution in user–computer interfaces. Hence, there are new opportunities to facilitate and simplify computer access, including the practical use of these interfaces in vast applications [4,5] and a broad spectrum of user communities. Designing models that can predict brain activity is an extensive research field, where one crucial aspect is feature selection, which is used to find the patterns that describe data.

At present, different techniques are used for feature selection, such as probability, logic, optimization, and so on, to solve various problems and to obtain the best features to solve a classification problem [6,7,8]. Having the most representative features for the description of the problem allows the classifier to converge to the correct solution. Deep learning [9] is a current group of techniques, within the range of black-box solutions, producing the most promising results. With deep learning, some architectures such as convolutional neural network (CNN) with different layers extract features from the image, and others perform classification within the same architecture. In some scenarios with large amounts of high-dimensional data, without considering the distributions and the correlations between features, overfitting problems can occur.

One possible solution is implementing a benchmark combined with a validation technique, such as *k*-fold cross-validation, to determine which models can solve a classification problem [10], the training of the models is usually run over a different data set, in order to obtain scores for different algorithms and choose the best one, according to the input data. This method is often impractical for larger numbers of hyperparameters, depending on the model used; for example, a natural question is: Which classification algorithm can be used to solve a particular problem? For this, we could use neural networks or a support vector machine (SVM) with linear or non-linear kernels. This is an essential decision, as one algorithm can converge to the solution faster than the others with similar accuracy, depending on the type of input data. Notably, these algorithms usually randomly initialize their parameters and cannot reproduce the same percentage of accuracy in each execution, even when using the same hyperparameters. In classification algorithm applications, we need to analyze the relation of the trained models and the input training data set to determine the complexity of the problem.

Many applications of machine learning and, most recently, computer vision have been disrupted by the use of CNNs [11,12,13,14,15]. Combining a minimal need for human design and the efficient training of large and complex models has allowed them to achieve state-of-the-art performance on several benchmarks. However, this performance is only possible with a high computational cost, due to the use of chains of several convolutional layers, often requiring implementations on GPUs or highly optimized distributed CPU architectures to process large data sets [16]. The increasing use of these networks for detection in sliding window approaches and the desire to apply CNNs in real-world systems mean that the inference speed has become an essential factor for various applications [16].

One significant problem is choosing the correct CNN architecture [17,18,19]. The sample size for training the CNN architecture usually is enormous, considering that it will depend on the election of filters and the rest of the hyperparameters in a CNN. Thus, transfer learning has been one of the most used techniques in the past years, implementing pre-trained architectures for classification [20,21]. This approach brings some advantages, reducing the processing time in training and reducing the amount of data required. One of the new challenges of this approach is choosing the sample size for training and the correct generalization of the features for new classification problems. Based on the above arguments, the most straightforward answer is that more data is needed to improve the results, and, in some scenarios, it is impossible to improve the results, thus leading to overfitting [22]. The sample size depends on the nature of the problem and the implemented architecture. Still, co-dependency can occur, making it necessary to test different architectures with appropriate data. Real-world data are never perfect and often suffer from corruption produced by noise, which may impact the interpretation of the training data and affect the performance of the model [23]. Additionally, noise can reduce the system performance regarding classification accuracy and training processing time. Existing learning algorithms integrate various approaches to enhance their learning abilities in noisy environments however, these approaches can have severe negative impacts.

The problem of learning in noisy environments [24] has been the focus in fields related to machine learning and inductive learning algorithms. In real-world applications, classifiers are developed and trained without a clear understanding of the noise factor. Thus, comprehensive knowledge of the noise in each data set avoids possible pitfalls during training. To overcome this type of problem, some approaches have experimented with different deep learning architectures, as they can extract features in their hidden layers without the need for human intervention however, this creates other problems. In deep learning, to compare the efficiency of different architectures with respect to the same problem [25], the benchmarks use different tests, such as cross-validation [10]. Even in this scenario, one of the problems with benchmark architectures is overkill. Due to the infinite number of possible architectures that can be proposed within the deep learning design area, it is impossible to test all of them. In addition, a large amount of information is needed to train this type of architecture. The larger the architecture, the higher the number of parameters that need to be trained. Due to this, techniques such as transfer learning [26] have become popular, utilizing the application of architectures that have been trained previously to solve new classification problems. These models can effectively serve as a generic model of the visual world, with different approximations [11,12,13,15].

Transfer learning takes advantage of these learned feature maps without having to start from scratch by training a large model on an extensive data set [26,27,28,29]. However, one of the main drawbacks of transfer learning is its dependency on heavy architectures, which are often excessive for specific problems. Thus, in this work, we introduce a series of texture amortization map features and a novel geometric classification score. These texture amortization map (TAM) features are based on the texture ideas that improve the generation of new features, with inherent adaptability according to the input data. The geometric classification score (GCS) is a score that can help to choose the best convolutional layers or a combination thereof, given a pre-trained architecture and a training data set for a specific classification problem.

## 2. Literature Review

In this section, the basic theoretical foundations are reviewed. First, we review some of the basic techniques in machine learning, such as feature generation and selection [6,7,8,30,31], and classification models [32,33,34]. Then, we review the basic CNN architectures [9,35].

### 2.1. Noise in Data Sets

Noisy data have a large amount of additional meaningless information called noise [23]. The noise in the data can be produced by many different sources, such as the error introduced in the instance attribute values, leading to contradictory examples [36]. As a case in point, noise can be created by the same examples having different class labels, where misclassifications can be seen as any data that a user system cannot understand and interpret correctly.

### 2.2. Class Ambiguity

Class ambiguity, in a classification problem, refers to the lack of discrimination in some classes using the given features by a classification algorithm [37]. Event-related potential (ERP) detection involves noisier data primarily as the features are intrinsically inseparable.

For each classifier, the processes of features extraction and selection can affect this ambiguity. Again, this operation represents the most informative item of the classes. Class ambiguity can occur for only some input cases. The classes are ambiguous for at least some cases and are said to have non-zero Bayes error [38], which sets a bound on the lowest achievable error rate.

### 2.3. Characterization of Geometrical Complexity

The geometric complexity of class boundaries is one of the most-studied [39,40] topics in the area of deep learning architectures in recent years. It is possible to define each classification problem as represented by a fixed set of training data, consisting of points in a *d*-dimensional real space $R^{d}$. A single set of training points are associated with their respective class labels. Furthermore, these problems work with the assumption of a sparse sample training set [41]. Thus, there are unseen points from the same source that follow the same (unknown) probability distribution, representing the data in the classification problem. Still, these unseen points are unavailable during the classifier design. These finite and sparse samples limit our knowledge about the boundary complexity. Thus, a measure that can describe the geometrical and topological characteristics of point sets in high-dimensional spaces over sparse data sets would be useful. Such a measure can provide a basis for analyzing the behavior of the classifier beyond the estimates of error tests. Some works have discussed several valuable measures for characterization in the analysis of data sets [39].

### 2.4. Interpretability of Convolutional Neural Networks

Convolutional neural networks [42,43,44,45] have achieved superior performance in many visual tasks, such as object classification and detection. Convolutional neural networks learn abstract features and concepts from raw image pixels [46]. This is why CNNs are also known as black box models; in the area of deep learning, model interpretability is a crucial issue. In [47], the following question was asked: “Can we modify a CNN to obtain interpretable knowledge representations in its convolutional layers?” Some studies have focused on trying to define interpretability, in terms of the input data. For instance, to define the curly concept, a set of hairstyles and texture images has been used [48]. In other examples, researchers have tried to answer the question: “Does a CNN learn semantic parts in its internal representation?” [49], by analyzing the response to the semantic parts of an object from the trained filters of a CNN.

The approach to making the learned features explicit is called feature visualization [50]. Feature visualization for a unit of a neural network is achieved by finding the input that maximizes the activation of that unit, as has been shown in several studies [47,51,52]. This process visualizes the features learned by the CNN architecture through activator maximization. For this, it is assumed that the parameters of a CNN are fixed once it has been trained. In that scenario, it is possible to search for a new image that maximizes the activation of each neuron. However, feature visualization cannot explain if the patterns are learned by the filters in the CNN. For this reason, the approach by [53] (network dissection) quantifies the interpretability of a unit of a convolutional neural network. It links highly activated areas of CNN channels with human concepts (objects, parts, textures, and colors). Network dissection has three steps:

- Obtain images with human-labeled visual concepts (e.g., from stripes to skyscrapers);
- Measure the CNN channel activations for these images; and
- Quantify the alignment of activation and labeled concepts.

However, much research remains to be conducted in order to fully understand which parts of the CNNs are involved in the classification tasks.

### 2.5. Transfer Learning

Transfer learning is a machine learning technique where a model trained for a specific task is reused in a second, related task [26]; for example, knowledge gained while learning to recognize cars can be applied when trying to recognize trucks. Thus, in recent years, this type of technique has become popular, mostly due to the accurate results that can be obtained, and as deep learning architectures have been increasing in size [35,54,55]. These architectures require large amounts of data, considerably increasing their training complexity. Transfer learning consists of two main components: A label space Y and an objective function f:X→Y. The function *f* can be obtained from any architecture (e.g., VGG-16 [35]), through exchanging the last layer with the new samples of the label space to be classified. Thus, the training is performed over the new samples to learn this new data set {X,Y}.

(1) $$T=\left\{Y,f(x)\right\},$$

given a source domain $D_{S}$ and learning task $T_{S}$, a target domain $D_{T}$, and learning task $T_{T}$, where $D_{S}\neq D_{T}$, or $T_{S}\neq T_{T}$. It is possible to transmit the knowledge learned from the domain $D_{S}$ and the task $T_{S}$ to the new objective function in $T_{T}$ [56].

## 3. Filter Approximation and Design

Filter banks have been widely used in computer vision for feature extraction. The purpose of a filter is to extract features and enhance the details of an image, even in the presence of noise. Therefore, such filters have been associated with the detection of sides or edges [57].

### Texture Filters

**Figure 1:**
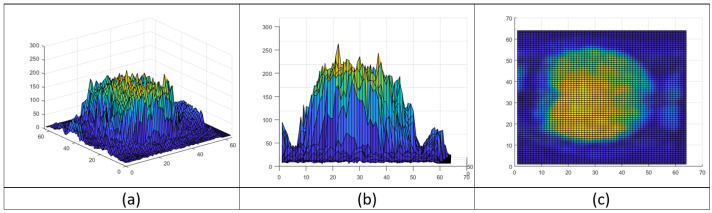
Topological map of brain activity obtained from fMRI. (**a**) Topological map of fMRI, (**b**) Side view and (**c**) Top view.

Traditional filter design techniques in computer vision have the objective to extract specific features, usually going through a process of smoothing to eliminate textures and preserve the shapes of objects to find other types of features. In this work, we will concentrate on filters design based on texture. For this, we will take sample data in a specific instant to visualize in Figure 1 where the information related to the texture in our data is represented in a topological map showing the distribution of the data points in our sample. It is possible to solve this classification problem from different perspectives. For example, we can try an approach using CNNs. However, this type of architecture needs a large amount of data and, for this specific problem, we have a limited number of samples. This small amount of samples may result in inadequate data distribution, given their sparsity. To overcome this problem, we propose kernels to adapt to the distribution of the pixels in the images from fMRI data. A kernel, also known as convolution matrices or mask, is a matrix that slides across the image and multiplies with the input. The output is enhanced in a desirable manner, extracting features that allow us to find the patterns to classify the response like in the fMRI data. This approach allows for increased control over the generated features.

For this, texture is one of the essential features used for identifying objects or regions of interest in any image [58], regardless if the image is a photomicrograph, an aerial photograph, or a satellite image.

**Figure 2:**
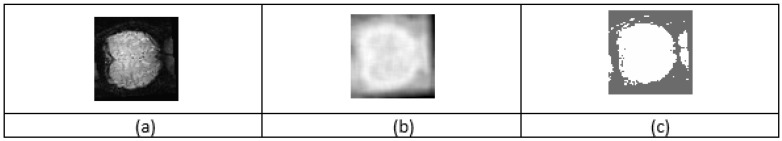
Comparison of texture maps. (**a**) Original slice of brain activity measure in fMRI, (**b**) Entropy map and (**c**) Contrast map.

In Figure 2, we compare diverse texture maps; for example, entropy and contrast. We can see that each map extracts different features inside the brain area of the same sample. The convolution works with the specific kernel of each texture, obtaining the texture response. Thus, we can analyze some features based on the texture of the brain activity and spatial information. For this, we need to define how we obtain these filters and feature maps from the fMRI samples. Thus, we define the TAM to extract the texture maps as follows (Equation (2)).

**Definition** **1.** *Let TAM(x,y) define a new image $I{\left(x,y\right)}^{\prime }$, in terms of an existing image I(x,y) in $R^{2}$, for all locations or coordinates (x,y)$\in R^{2}$ = (−∞,∞) as follows:*

(2) TAM(x,y)=T(x,y)(K∗I(x,y))=T(x,y)∑∑K(u,v)I(x−u,y−u),

*where K is the kernel, I(x,y) is the input image, and T(x,y) is the texture measure of the image at position (x,y).*

To obtain the texture maps from TAM(x,y), we need to ensure that the input and output behavior are independent of the specific spatial locations. This property is called shift-invariant (or space-invariant) [59].

The texture in images can be evaluated from different measurements that provide different insights; based on different works analyzing textures in medical images [60,61,62]. Contrast, homogeneity, entropy, and energy were used in the work. The implementation and objective of the expected features are described in detail in this chapter.

Thus, we can choose a texture function to convolve with the filtered image, where T(x,y) in $R^{2}$ is a transformation from $I{\left(x,y\right)}^{\prime }$ in $R^{2}$. This provides us with the transformation of the obtained filtered image. Thus, to measure the randomness of the pixel distribution with respect to length or orientation, we measure the entropy of the image, so as to take a higher value for a more random distribution measuring the amount of disorder in the image (Equation (3)) [63]:

(3) E=−∑∑p(i,j)log(p(i,j)).

**Figure 3:**
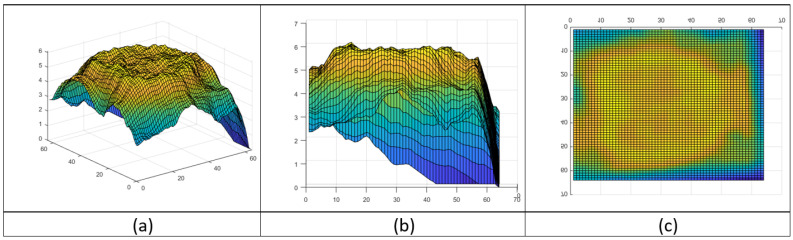
Brain activity topological entropy map. (**a**) Topological map of entropy, (**b**) Side view and (**c**) Top view.

Entropy maps are essential, as they produce a map of the possible configurations of the brain activity, as shown in Equation (3). These maps measure some noise from the images, but also evaluate the entropy. These differences in the features allow a machine learning pipeline to identify the patterns for the classification task. Thus, visualization of the topological entropy map (Figure 3) shows the detected activity in the brain response in the fMRI. To obtain the feature map of the entropy, we substitute T(x,y) with Equation (3) in TAM(x,y) (Equation (2)), obtaining Equation (4):

(4) $$\begin{array}{cc} TAM_{E}\left(x,y\right) & =\\ \; & =T_{E}\left(x,y\right)\left(K*I\left(x,y\right)\right)\\ \; & =-\frac{\sum \sum p(i,j)log(p(i,j))}{MN}\left(K*I\left(x,y\right)\right). \end{array}$$

To continue the extraction of different feature maps, we measure the contrast to determine the sample differences in color and brightness, in order to obtain the contrast [64] in an image (Equation (5)):

(5) $$Contrast=\sum \sum n^{2}p\left(i,j\right).$$

Taking the expression of Equation (5) and substituting for T(x,y) in TAM(x,y) (Equation (2)), we obtain the feature map of the contrast,    

(6) $$\begin{array}{cc} TAM_{C}\left(x,y\right) & =\\ \; & =T_{C}\left(x,y\right)\left(K*I\left(x,y\right)\right)\\ \; & =-\frac{\sum \sum n^{2}p\left(i,j\right)}{MN}\left(K*I\left(x,y\right)\right). \end{array}$$

Now, in contrast, we measure the difference in the distribution of the pixels. We also obtain the homogeneity, which represents the gray-level distribution of the pixels in the image sample, regardless of their spatial arrangement. With this measure, we can compare the homogeneity changes. Thus, to obtain the homogeneity feature map, we use the following definition [65]:

(7) $$Homogeneity=\sum \sum \frac{1}{1+n^{2}}p\left(i,j\right).$$

Then, to compute the feature map of the homogeneity, we take Equation (2) and substitute T(x,y) with Equation (7) to obtain Equation (8):

(8) $$\begin{array}{cc} TAM_{H}\left(x,y\right) & =\\ \; & =T_{H}\left(x,y\right)\left(K*I\left(x,y\right)\right)\\ \; & =\sum \sum \frac{1}{\left(1+n^{2}\right)\left(MN\right)}\left(K*I\left(x,y\right)\right). \end{array}$$

The homogeneity feature map provides a uniform measure of the composition of the fMRI data textures. In this analysis, we also focus on the energy calculation, as it is used to describe a measure of information. The energy feature map corresponds to the mean squared value of the signal (typically measured based on the global mean value) [66]. This concept is usually associated with Parseval’s theorem [67], which allows us to consider the total energy distributed among frequencies. Thus, one can say that an image has most of its energy concentrated in low frequencies. To obtain the feature map of the energy, we use:

(9) $$Energy=\sqrt{ASM},$$

with ASM defined as (Equation (10)),

(10) $$ASM=\sum \sum p{\left(i,j\right)}^{2}.$$

Thus, to compute the feature map of the energy, we take Equation (2) and substitute T(x,y) with Equations (9) and (10),

(11) $$\begin{array}{cc} TAM_{P}\left(x,y\right) & =\\ \; & =T_{P}\left(x,y\right)\left(K*I\left(x,y\right)\right)\\ \; & =\frac{1}{\left(MN\right)}\left(\sqrt{\sum \sum p{\left(i,j\right)}^{2}}\right)\left(K*I\left(x,y\right)\right). \end{array}$$

## 4. Geometric Classification Score (GCS)

In the present study, we work with different CNN architectures to optimize the selection of features obtained from the convolutional layers. This analysis helped us to understand what is happening during the training stage or inside a trained CNN. Not all the convolutional layers or combinations thereof are the best transformations for the input data. Sometimes, the aim is to identify which convolutional layers should be kept and which should be removed, in order to obtain the best representation of the data.

**Figure 4:**
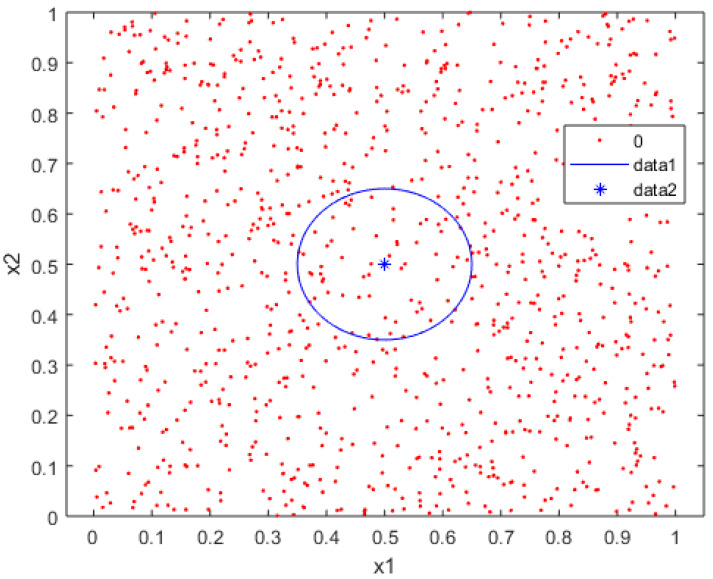
Outlier observation with the range of their neighborhood.

For this, we can use the concept of an outlier. An outlier is defined as an observation extremely far from the main set of observations. Thus, we need to identify and remove them [68]. Outliers are frequently a source of noise in the data, thus affecting the classification problem. We know that a point or set of points creates noise in the classes when they are mixed under some parameters in the nearby class, as shown in Figure 4.

**Figure 5:**
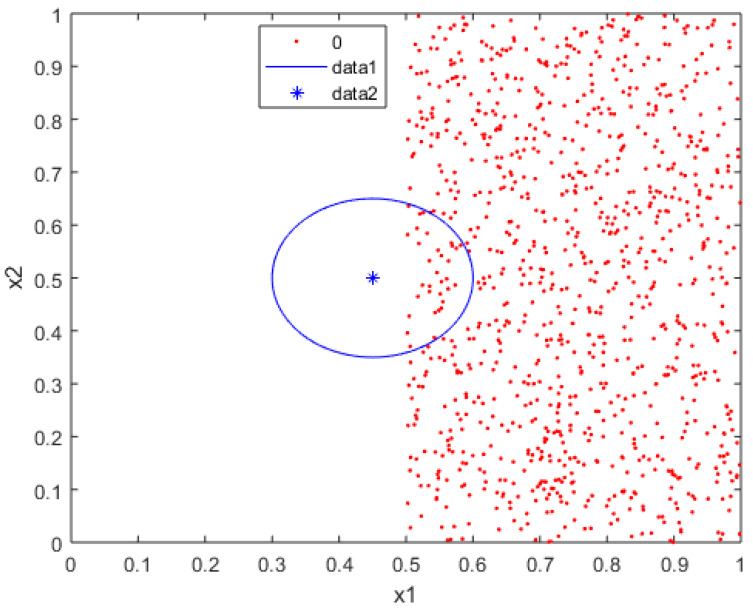
Points of observation with the range of their neighborhood.

An outlier will not necessarily invariably be defined as a noise point. In some cases, these outliers are intrinsic to the phenomenon being modeled with the data. Furthermore, in several instances, an outlier can be classified without any significant issues, as shown in Figure 5, which shows how the blue point that is far from the rest of the data set is considered an outlier however, it is not surrounded by points of the red class, which means that the data can be linearly separated, even though an outlier is near the red class.

A crucial step is defining if the point in the map is noise, which directly depends on some parameters that we use to measure the amount by which the classification is affected. Remember that some problems are classified as linearly separable or non-linearly separable data. We can analyze whether a point is noise, concerning the other class. For this, we take a sample point of the blue class and draw a radius to analyze the points of the red class within that neighborhood, delimited by the circle shown in Figure 5. We can see that the blue point is an outlier, and the other class points do not surround it. This means that we can draw a line between these data to achieve classification, and not consider this point as noise.

Let us consider a finite data set of *M* samples with *N* features $A_{M,N}=\left\{x_{1},x_{2},\ldots ,x_{M}\right\}$ and a set of hypotheses h∈H, such that h:A→{0,1}. We can consider the data set as a multi-class problem [69], when we have *K* classes for which there are subsets of $A_{M,N}=\left\{C_{1},C_{2},C_{3},\ldots ,C_{K}\right\}$. If we want to determine if a class $C_{k}$ is noise, with respect to the rest of the classes in the data set, we can extract class $C_{k}$ from $A_{M,N}$ to form an M−tuple to compare, with respect to $C_{k}$. In this scenario, we can consider the problem as a dichotomy. As such, we need to define a measure, GCS, to determine if $C_{k}$ is noise concerning the new M−tuple.

**Proposition** **1.** *The GCS measures the number of noise points present in class one with respect to class two, classifying each noise point of class one according to the number of points in class two that surround each point.*

**Construction** **1.** *To have a dichotomy generated by* H *on the set of points in* $A_{M,N}$ *, we extract class* $C_{k}$ *to measure the number of noise points with respect to the rest of the points. As such, we obtain the subset* $B_{k}$ *(Equation (12),*

(12) $$B_{k}=\underset{j\neq k}{⋃}C_{j}\;\;\forall \;j=1,2,\ldots ,K,$$

where we can test if each point from subset $C_{k}$ is noise with respect to the subset $B_{k}$. So, we take a point from $C_{k}$ that is the center of an *n*-sphere. An *n*-sphere (or *n*-hypersphere) is a topological space that is homeomorphic to a standard *n*-sphere [70]. It is a set of points in (n+1)-dimensional Euclidean space situated at a constant distance *r* from a fixed point. The *n*-hypersphere is a generalization of a circle (or a 2-sphere); for example, the usual sphere is a 3-sphere [71]. For dimensions n≥4, we can define a sphere as a set of n−tuples of points $\left(x_{1},x_{2},\ldots ,x_{n}\right)$, such that:

(13) $$x_{1}^{2}+x_{2}^{2}+\ldots +x_{n}^{2}=R^{2},$$

where *R* is the hypersphere radius and represents the constant distance of its points to the center. In terms of the standard norm, the *n*-sphere is defined as in Equation (14),

(14) $$S^{n}=\left\{x\in R^{n+1}:\left\|x\right\|=1\right\},$$

and an *n*-sphere of radius *r* can be defined as:

(15) $$S^{n}\left(r\right)=\left\{x\in R^{n+1}:\left\|x\right\|=r\right\}.$$

The *n*-dimensional sphere is the surface or boundary of an (n+1)-dimensional ball.

Topologically, an *n*-sphere can be constructed as a one-point compactification of *n*-dimensional Euclidean space [72]. Briefly, the *n*-sphere can be described as $S^{n}=R^{n}\cup \left\{\infty \right\}$, which is an *n*-dimensional Euclidean space plus a single point, representing infinity in all directions [70]. In particular, if a single point is removed from an *n*-sphere, it becomes homeomorphic to $R^{n}$. This constitutes the basis for stereographic projection [73]. When working with a Euclidean space, we denote it by $E^{n}$. If $x\in E^{n}$, then x has the shape of $x=(x_{1},x_{2},\ldots ,x_{n})$ with $x_{i}\in R$, and $E^{n}$ is designated an inner product, given by:

(16) $$\begin{array}{cc} \; & E^{n}\times E^{n}⟶R\\ \; & \left(x,y\right)⟶x\cdot y=\sum_{i=1}^{n}x_{i}y_{i}. \end{array}$$

Now, consider the following geometric idea: When taking two points $x_{1},x_{2}\in E^{n}$ and performing a subtraction, we obtain the vector *l* that passes through $x_{1}$ and $x_{2}$. If we take any other point *x*, such that $l^{\prime }=x-x_{2}$ is perpendicular to *l*, we obtain the set $P=\{x|\left(x_{1}-x_{2}\right)\cdot \left(x-x_{2}\right)=0\}$, which allows us to separate the Euclidean space $E^{n}$ [74]. The Euclidean coordinates in (n+1)-space, $\left\{x_{1},x_{2},\ldots ,x_{n+1}\right\}$, that define an *n*-sphere $S^{n}$, are represented by Equation (17),

(17) $$r^{2}=\sum_{i=1}^{n+1}{\left(x_{i}-c_{i}\right)}^{2},$$

where $c=(c_{1},c_{2},\ldots ,c_{n+1})$ is the center point of the *n*-sphere with radius *r*. To calculate the GCS, the point $\vec{v}$ in $B_{i}$ is the center in Equation (17). The *n*-sphere (Equation (17)) exists in (n+1)-dimensional Euclidean space and is an example of an *n*-manifold. The volume, ω, of an *n*-sphere of radius *r* is given in Equation (18) [73]:

(18) $$\omega =\frac{1}{r}\sum_{j=1}^{n+1}{\left(-1\right)}^{j-1}x_{j}\max dx_{j}=*dr.$$

Thus, we create a subset of all the points inside each *n*-sphere measuring the Euclidean distance dist of the point $\vec{v}$ in $B_{i}$, concerning all points $\vec{w_{j}}$ in $C_{i}$, to obtain the new subset $M_{i}$ (Equation (19)),

(19) $$M_{i}=\left\{\left(\vec{w_{j}}\right)\;\;\forall i=1,2,\ldots ,N\;\;\forall \vec{w_{j}}\in C_{i}|dist\left(\vec{w_{j}},\vec{v}\right)<r\right\}.$$

With this new subset, we obtain the subset of the closest neighbors to the center of the *n*-sphere, in order to define whether the point will be labeled as noise or not. As we are using an approximation of the coverage of all the points of this new subset $M_{i}$, having the geometric shape of an *n*-sphere, which does not describe the real geometric shape of the subset of points, the next step is to obtain the polytope that covers all the points of the subset. The affine envelope of a subset $D\subset E^{n}$ is the smallest affine space containing *D*. Thus, if we have a set of points *S* in $E^{n}$, *S* is convex and, for any two points $x_{1},x_{2}\in S$, we have a line segment connecting them inside the convex set:

(20) $$\bar{x_{1}x_{2}}=\left\{\lambda x_{1}+\left(1-\lambda \right)x_{2}\;|\;0\leq \lambda \leq 1\right\}.$$

By definition, a polytope is the convex hull of a finite non-empty set in $R^{n}$ [75]. Thus, a polytope is the convex hull of a given set of points $P=\{p_{1},\ldots ,p_{m}\}$. Algebraically, ${\left||X|\right|}^{2}=X^{T}X$ must be minimized for all *X* of the form in Equation (21),

(21) $$X=\sum_{k=1}^{m}P_{k}w_{k},\sum_{k=1}^{m}w_{k}=1,\;\forall \;\;w_{k}\geq 0.$$

If $k_{1},\ldots ,k_{n}$ are convex sets, then ${⋂}_{i=1}^{n}k_{i}$ is convex. To see this, consider two points $x_{1}$ and $x_{2}$ in ${⋂}_{i=1}^{n}k_{i}$. Since any $k_{i}$ is convex, the line segment $\bar{x_{1}x_{2}}\in k_{i}\;\forall \;i$. Thus, ${⋂}_{i=1}^{n}k_{i}$ is convex. The convex hull conv(k) of $k\subset E^{n}$ is the smallest convex hull containing the points, in view of:

(22) $$conv\left(k\right):=⋂\left\{k^{\prime }\subset E^{n}\;|\;k\subset k^{\prime }\;with\;k^{\prime }convex\right\},$$

the convex hull of a finite set $U=\left\{u^{1},\ldots ,u^{n}\right\}\subset E^{n}$ of points; that is, the set has the form in Equation (23),

(23) $$conv\left(\left\{U\right\}\right)=\left\{\sum_{i=1}^{N}\lambda_{i}\vec{u_{i}}|\lambda_{i}\geq 0,\sum_{i}^{N}\lambda_{i}=1\right\}.$$

Accordingly, the convex hull is a finite set of points $u_{1},u_{v},\ldots ,u_{n}$, which can be written as a convex combination [76]:

(24) $$C=\left\{\;\lambda_{1}u_{1}+\ldots +\lambda_{n}u_{n}\;|\;\sum_{i=1}^{N}\lambda_{i}=1\;and\;\lambda_{i}\geq 0\;\forall \;i\right\}$$

and

(25) $$x=\sum_{i=1}^{N}\lambda_{i}u_{i},\;\;y=\sum_{i=1}^{N}{\lambda_{i}}^{\prime }u_{i}\in C.$$

With the convex combination, we generate a polytope from the subset $M_{i}$, and we can evaluate whether the point $\vec{v}$ is inside the polytope, to consider it (or not) as a noise point, where $NS_{k}$ is the sum of the number of noise points for class *k*. We repeat that process for all points in all the classes, as described in Algorithm 1. With this proposal, GCS allows us to identify if a point in a data set is classifiable or not; finally, it is defined as Equation (26):

(26) $$GCS_{k}=1-\frac{|NS_{k}|}{|C_{k}|}\;\;\forall \;k=1,2,\ldots ,K..$$

**Algorithm 1** Algorithm to obtain the GCS of a data set.**Input: **$A_{M,N},radius$**Output:** score**Method:**noiseCounter = 0Normalize $A_{M,N}$**for** each $x_{i}$ in $A_{M,N}$ **do**   **for** j=0 to *M* **do**     **for** i=0 to *N* **do**        subset={}        $distance=(x_{i}\left[j\right],B\left[i\right])$        **if** distance<radius **then**          $subset.append(x_{i}\left[j\right])$        **end if**     **end for**     Obtain convex hull(subset)     **if** Point $x_{i}\left[j\right]$ is in convex hull: **then**        noiseCounter++     **end if**   **end for****end for**Obtain score from noiseCounterreturn score

Obtaining a measure such as the GCS provides several possibilities, as well as questions to answer. For example, as mentioned in the Introduction, we know that many of the applications of deep neural networks particularly CNN (see, e.g., [77,78,79]) depend on the original architecture being trained with large amounts of data. These architectures trained with large amounts of data sometimes need special hardware to handle this amount of information, in order to process and train the architecture [20,21]. The question is then not related to the amount of data or the hardware, but rather whether each layer within the CNN provides valuable information for classification. This leads to another question: Can the architecture be optimized? The typical approach is to use benchmarks to evaluate the performance of the architectures when classifying, but that does not describe if the architecture’s hidden layers are efficient. Thus, we can ask if the GCS can help us in this task, by measuring the level of data classification according to each transformation for each hidden layer within the deep learning architecture.

A deep architecture such as a CNN has the advantage of being able to generalize the rules that characterize the classification problem being solved. Obtaining the set of rules that describes this process is not new; it has been investigated in other areas, such as the Vapnik–Chervonenkis theory [80], where the objective is to identify the rules that can be generated within the hidden layers of a classification system and determine if this set of rules is transmitted in each layer. Thus, we need to continue and define shattered sets.

**Definition** **2.** *Let X be a set and Y a collection of subsets of X. A subset A⊂X is shattered by Y if each subset B⊂A of A can be expressed as the intersection of A with a subset T in Y. Symbolically, then, A is shattered by S if, for all B⊂A, there exists some T⊂X for which T⋂A=B. If A is shattered by Y, then Y shatters A if [81]:*

(27) P(A)=T⋂A:T∈Y,

*where P(A) denotes the power set of A, in the field of machine learning theory.*

We usually consider the set *A* to be a sample of outcomes drawn according to a distribution *D*, with the set *Y* representing a collection of known concepts or laws. In this context, we can see the outputs of each hidden layer of a deep learning architecture as different sets of possible rules, if we combine it with transfer learning techniques. Thus, if we obtain the GCS measure in each layer, we obtain the best output candidate to solve the classification problem. We can consider this output as a set of rules *A*, where *A* is shattered by *Y*. As such, the set *Y* can explain the new rules obtained by solving the same job and optimizing the architecture.

**Hypothesis** **1** **(H1).** *Suppose we assume that all the data transformations in the inner layers of a deep neural network still belong to a Euclidean space. In that case, we can evaluate the data transformations inside the hidden layers and determine how classifiable they are, in order to evaluate which are the best transformations of a CNN, for which Algorithm 2 is proposed. We can continue exploring this type of transformation for future work and begin to observe even manifolds that preserve belonging to a Euclidean space.*

To analyze the representation of the data in each hidden layer of a trained CNN, Algorithm 1 can be used to obtain the GCS measure of each layer. As such, we can compare which layer or combination of layers can extract the best features in classification problems. Thus, we can better understand the number of layers that do not contribute new information to the solution. As soon as we obtain the GCS measures within the hidden layers of a CNN architecture, we know which layers to remove and analyze and which layers are generalizing the rules learned in the training stage.

In this section, we describe the two proposals of this work. First, we present the case where, due to the nature of the type of fMRI data, traditional computer vision filters do not obtain the best results. We observed that traditional filters attenuated the patterns to be recognized. Thus, we propose TAM, which is an amortized feature filter, depending on the neighborhood of the pixels when extracting features and enhancing the patterns that we are looking for, in order to solve the classification problem. Finally, we use deep learning architecture techniques to help us classify these data, using TAM processing for these classification problems. Still, we identified another problem: This type of architecture has too many parameters to be trained. Sometimes, some layers do not contribute to solving the classification problem; conversely, in some cases, some layers may even introduce noise. Thus, we propose the novel GCS measure, which allows us to analyze the behavior of the transformations within the hidden layers and provides a measure that allows us to identify how classifiable the data set is in each hidden layer, thus allowing us to identify which layers can be removed. Therefore, in the following section, we present the results and analysis of testing the techniques mentioned in this section under different scenarios.
**Algorithm 2** GCS cut**Input: **trainData,testData,dnnLayers,dnnWeights**Output: **errorRate**Method:**Build DNN architectureLoad weights from DNN architecture**for** layer *l* in dnnLayers **do**   Get $GCS_{li}$**end for**Obtain max score in $GCS_{li}$ to get idLayerCut DNN in idLayerAdd multiclass layer to obtain dnnCutTrain dnnCut with trainDataObtain errorRate with testData in dnnCut

## 5. Results

**Figure 6:**
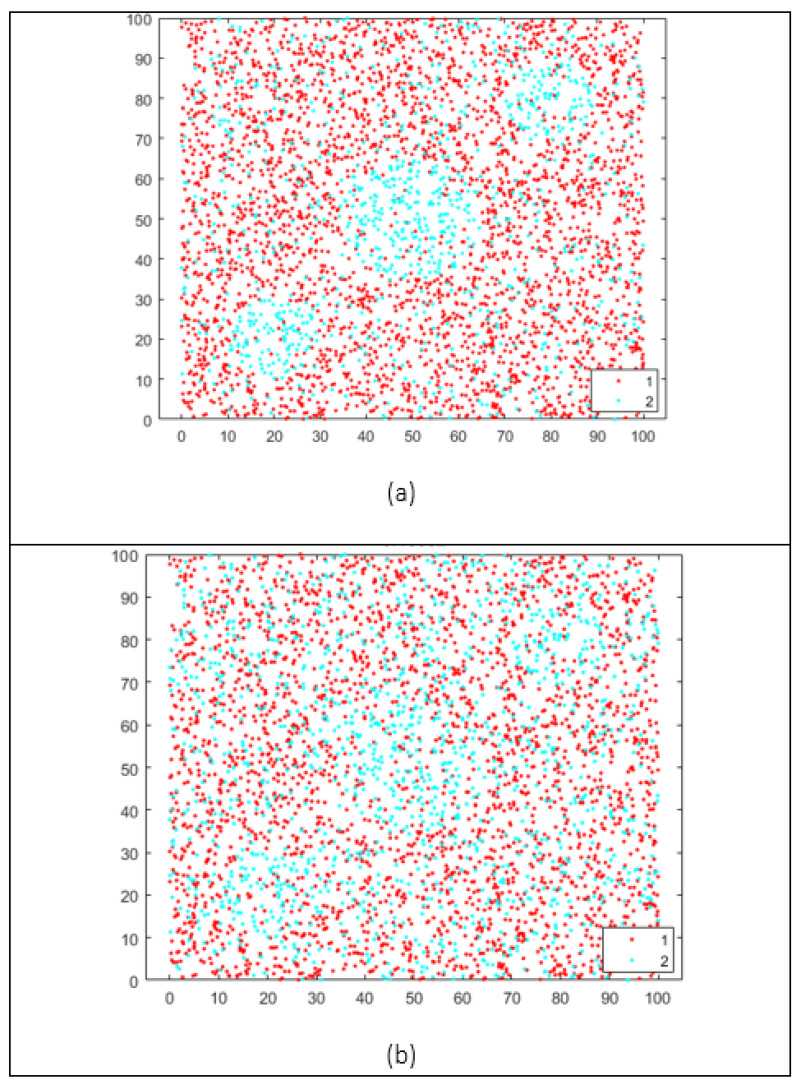
Non-linearly separable data examples. (**a**) Non-linearly data with low random noise and (**b**) Non-linearly data with high random noise.

In (Figure 6), we show a comparison between different data set examples. Different scenarios have the same data, but various amounts of noise points. In this scenario, we tested how classifiable a data set is through the score obtained from the GCS. We compare these data sets, including examples of linearly separable or non-linearly separable data, in order to analyze the behavior of the GCS. The GCS provides a value between −1 and 1. Any value close to one, regardless of the sign, indicates how classifiable a data set is, and the sign tells us if it is a linearly separable set or not. Otherwise, a value of zero indicates that the data set is not classifiable. In this example (Figure 6), we can observe cases where there is no added noise, as the GCS is close to 1 or −1. As we add noise to the data set, the GCS value approaches 0. In this way, the GCS can tell us how classifiable the data set is.

We have now proven that the GCS provides a measure for estimating classification accuracy. A data set may or may not have some data transformation, depending on the pipeline that is being applied in data processing. We analyzed a deep learning architecture scenario, as described in the previous section. We know that, between each hidden layer, the input data suffer from data transformation. This led us to evaluate the effect of each data transformation in each hidden layer, through the score obtained from the GCS. Considering our initial hypothesis for the GCS, we assumed that the hidden layer transformations keep the data in Euclidean space. As such, with GCS, we know which layers help to solve the classification problem and which harm this training process. In order to obtain the results in this work, we conducted four experiments, as presented in the following subsections.

### 5.1. MNIST Data set Classification

**Figure 7:**
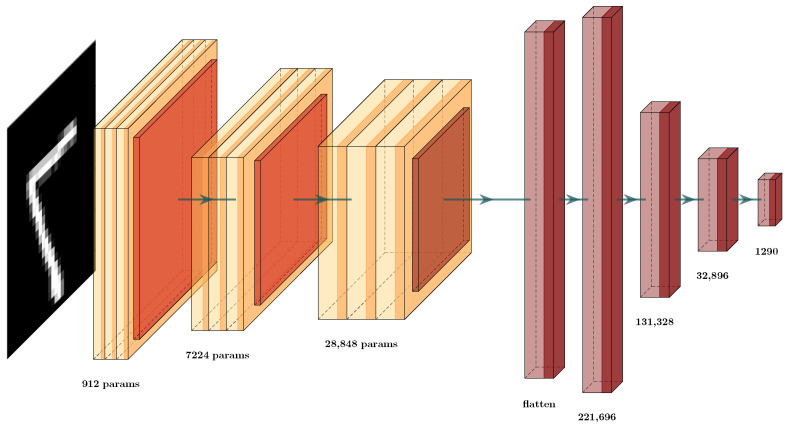
Convolutional Neural Network (CNN) architectures for MNIST classification (CNN (a)).

We tested the MNIST [82] data set, due to its comprehensive use in state-of-the-art methods. In addition, a CNN architecture was designed, in order to analyze the behavior of GCS (Figure 7), where a convolutional network was defined with the classic convolutional, max-pooling, and fully-connected layers. As previously mentioned, the convolutional layers possess a composition of different filters defined locally, which are optimized by the training process (Figure 7). This is achieved through the well-known back-propagation process, through the use of automatic differentiation [11]. We know that the aim of the convolution layers is to extract high-level features. This is why we obtain the GCS in the elements inside this architecture, in order to compare the behavior of each type of layer and determine how to optimize this architecture.

During training, these parameters/weights are changed, given the updates by the forward and backward procedures of training. Thus, the convolutional layers act as modifiable filters, extracting features first from a low level of interpretation to higher levels of interpretation. Thus, we had a GCS function at each of these layers, in order to score how well the new features are separable. The aim of this was to score the layer’s importance during the learning procedure. This allows us to decide whether or not to prune particular layers of the deep neural network (DNN). For this, we combined the ideas of transfer learning, using the GCS measure for each layer of the architecture.

**Figure 8:**
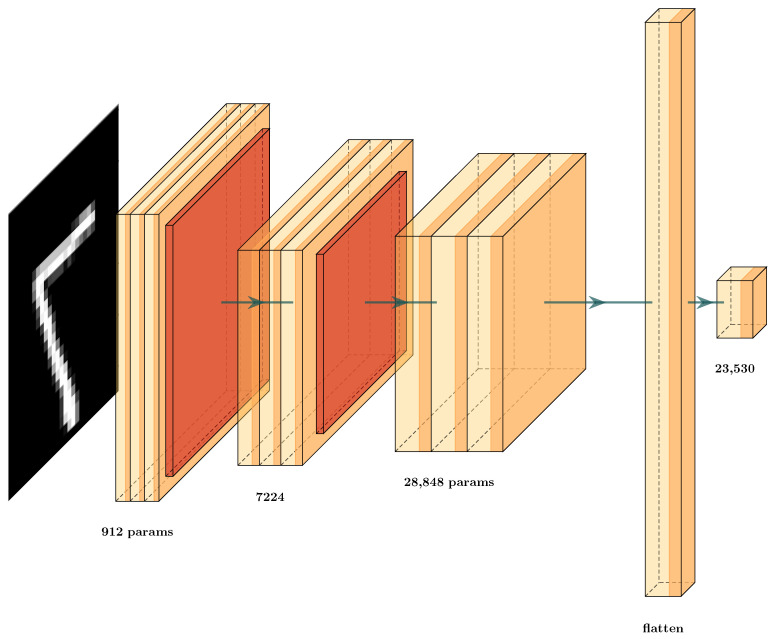
CNN architecture after using the Geometric Classification Score (GCS) cut Algorithm 2 (CNN-GCS (b)).

First, we trained the architecture (Figure 7) and used the GCS score to decide which layers are important in the classification effort to produce the GCS measure. This allowed us to identify which layers provided the best transformations during data processing and training. After training, we obtained the GCS score for each layer (Table 1 and Table 2). Based on those scores, we decided to prune certain layers. In the example in Figure 8, we can see the new architecture and how the GCS score helped to decrease the complexity of the layers of the DNN. This new architecture had a similar structure to that shown in Figure 7. Here, we see that the layers in red were removed, and the retained layers were those with a GCS measure value close to 1 or −1. For example, we compare the results obtained in Table 3, where the number of parameters in the original architecture CNN (a) was 424,194 weights. After GCS architecture reduction, the number the parameters decreased to 60,514. Thus, we used only 14% of the original architecture parameters however, even with this smaller architecture, the testing error decreased from 3.5% to 2.8%. We conclude that the GCS score can help to reduce the number of parameters while maintaining the performance of the original architecture.

**Figure 9:**
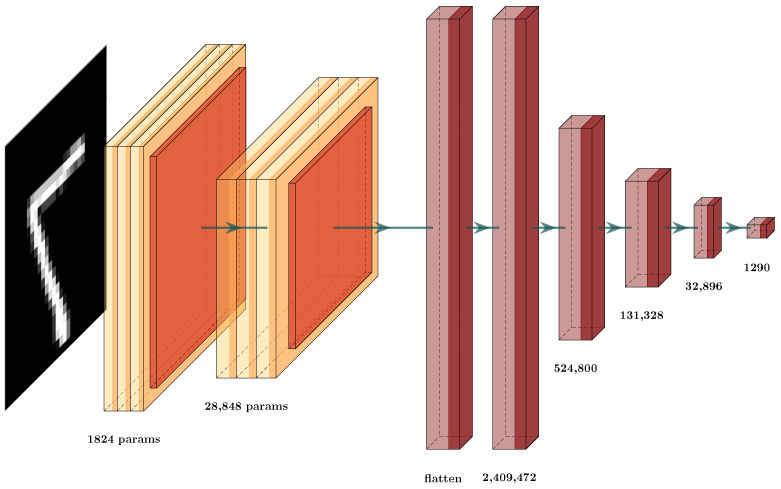
Second CNN architecture proposal for MNIST classification (CNN (c)).

**Figure 10:**
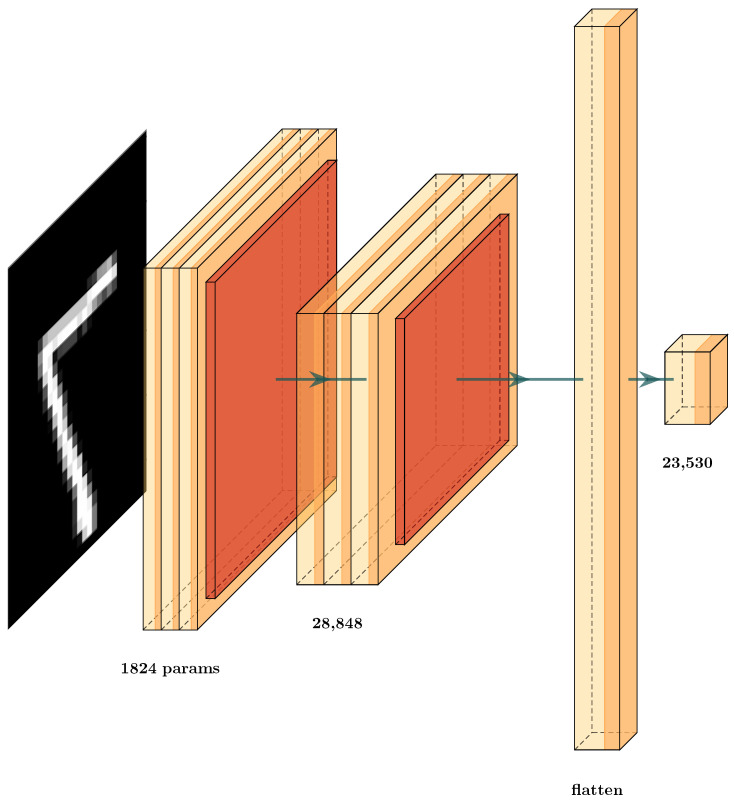
Second CNN architecture proposal after using the GCS cut Algorithm 2 (CNN-GCS (d)).

Now, in more detail, we examine the two architectures used to prove this hypothesis (Figure 7 and Figure 8). In Table 3, we display the testing error of each architecture on the validation set. The column Testing Error (%) shows the results for these architectures; in row CNN (a), the testing error and number of parameters for the original CNN (Figure 7) are given while, in row CNN-GCS (b), we see the results of the architecture after the GCS architecture reduction, as shown in Figure 8. To test whether this result supported our hypothesis, we experimented with an architecture similar to the first one in Figure 9, by changing the number of neurons in the dense layers. These results can be observed in row CNN (c); by applying the GCS architecture reduction through Algorithm 2, we optimized this architecture (Figure 10) to obtain the results in row CNN-GCS (d), where we can see a reduction in the number of parameters from 3,130,458 to 54,202. Basically, 98% of the parameters were eliminated, and testing error decreased from 2.5% to 2.4%. Although the testing error percentage slightly decreased, the number of parameters was considerably reduced.

The previous examples showed how GCS architecture reduction can improve the performance of deep architectures. This is astonishing, as the new architectures have considerably fewer parameters, even when compared to other state-of-the-art similar-sized architectures [83,84]. Table 4 shows how the GCS score improved the architecture and the performance; we compare the accuracy of our architectures after GCS architecture reduction (Table 4, columns CNN-GCS (b) and CNN-GCS (d)). We observed that, for the MNIST classification problem, when comparing our testing error to that of the other approaches, our error was higher by approximately 2% and, within the two proposed architectures, there was a variation of 0.4%. Compared to the others, this resulted in about 2 million fewer parameters, and the difference in testing error was very low however, the objective of using the GCS is optimization of the deep neural network architecture. Thus, we observed that the architectures were optimized by removing several layers of the proposed architecture. Notably, the proposed architectures were trained from scratch completely in the MNIST data set only, and the reduction was performed using the GCS score. This is different from the other architectures, as they are trained on much larger data sets [84]. Then, transfer learning was performed in the MNIST, which is an advantage, as more general filters are generated on the larger data sets, allowing them to perform better. After several failed attempts to obtain those data sets, we decided to use only the MNIST data sets. Thus, this is the reason for the difference of 1% or 2% between their architectures and our reduced architectures however, even under those restrictions, we believe that the GCS-reduced architectures are able to perform well, compared to larger architectures. This is important for intelligent environments, where resources are scarce [26].

In this subsection, we show that architecture optimization by GCS reduces the number of parameters and, in some cases, also reduces the error on the testing set. Thus, in the following sections, we propose combining the improved TAM filters, deep learning, and GCS score to improve classification in the ERP data set.

### 5.2. Deep Learning Architecture Optimization by GCS for ERP Detection

**Figure 11:**
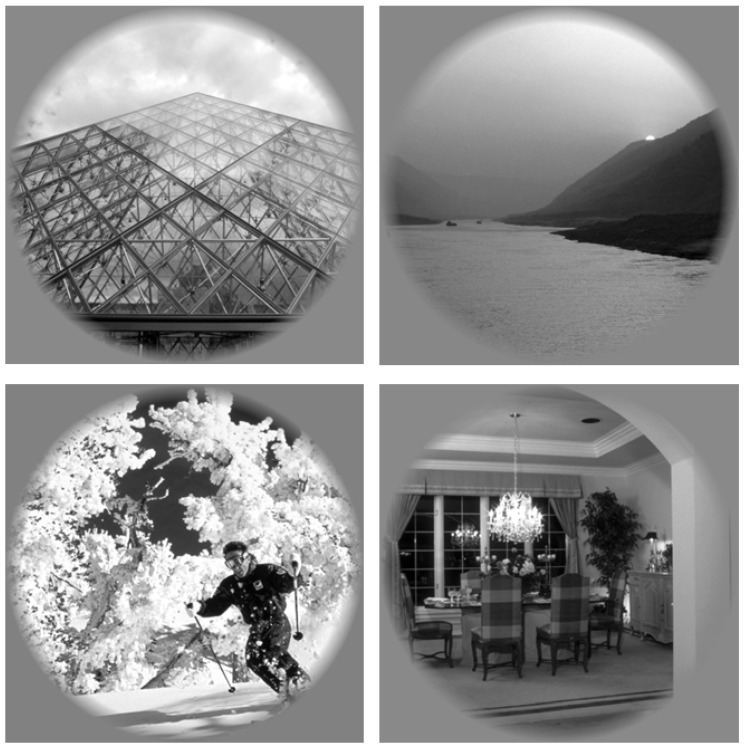
Samples input images such as people, animals, landscapes, etc. for the generation of the functional magnetic resonance imaging (fMRI) data.

**Figure 12:**
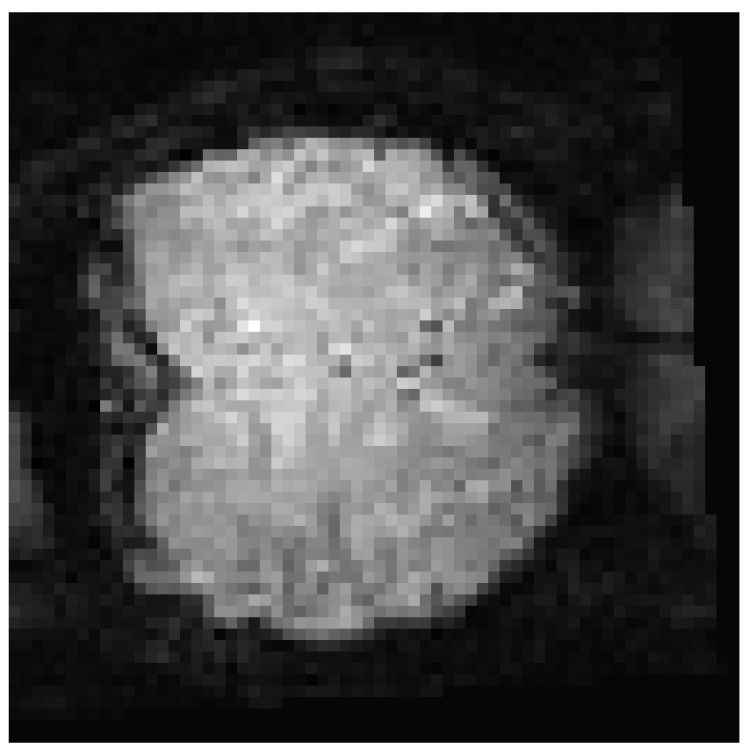
Sample layer of BOLD fMRI data of 64 × 64 pixels.

In this subsection, we present a classification problem for ERP measurement using fMRI data. This data set is composed of BOLD fMRI records of individuals with specific labeled images [85]. These images (Figure 11) show different scenarios, such as people, animals, landscapes, and so on. Thus, the data set was composed of the fMRI record together with the image and correct labeling of that image. Specifically, the data obtained from the BOLD fMRI recording produced by the ERP brain response generates a data cube that provides 18 layers of images, where each image (Figure 12) is a slice of the brain with dimensions of 64 × 64 pixels. This cube, with dimensions of 64 × 64 × 18, is the result of scanning the brain activity through the entire experiment described in [86].

**Figure 13:**
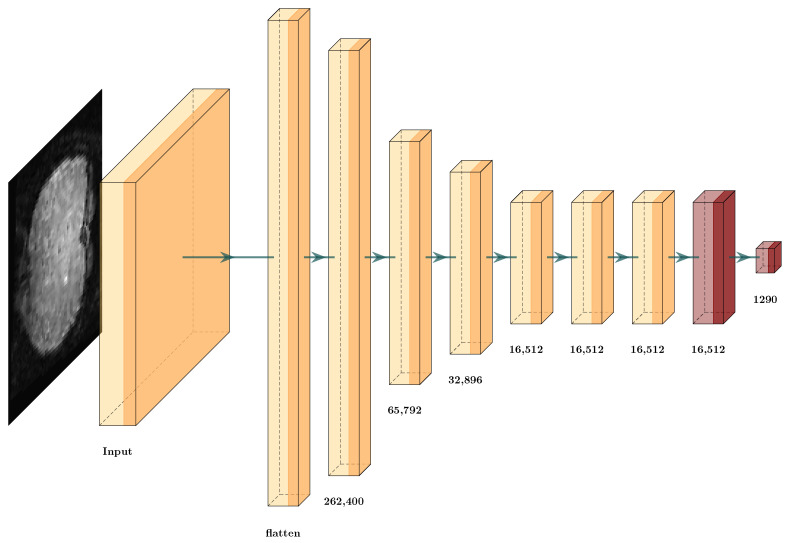
Architecture proposal from fMRI data for event-related potential (ERP) classification.

We propose the full integration of TAM and the GCS score in a deep learning architecture. First, we designed a simple deep learning architecture with the input 3D data and eight dense layers (Figure 13). This architecture obtains the cubes from the fMRI. Then, it uses the architecture to solve the ERP classification problem.

Table 5 lists the accuracy results obtained from the proposed architecture with a testing error of 23.05%, which is unsatisfactory. Here, we had two options: Change the input samples from the original data set for TAM features or reduce the proposed architecture using this methodology. We obtained the results in Table 6 for the GCS score. Even when the values from the layers obtained a GCS measure close to 1 or −1, we could not assure that the classifier would obtain 100% accuracy, as DNNs have different possible architectures with several different hyperparameters that provide different values in the prediction. Observing the results, we found that the value increased slightly as the number of layers increased until layer seven, where the value decreased. From this, we inferred that each transformation until the seventh layer helped to improve the classification performance of the CNN. Thus, we propose using the algorithm for the GCS score Algorithm 2 to select the correct set of layers for GCS architecture reduction.

**Figure 14:**
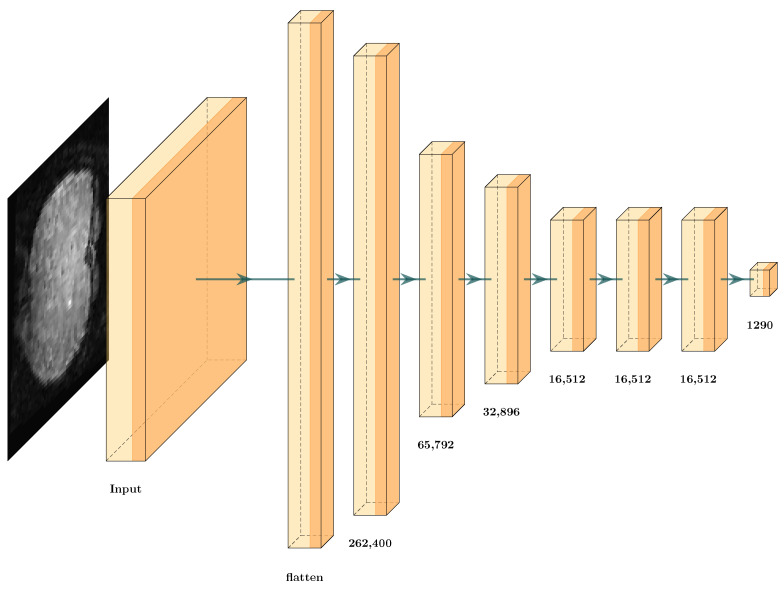
Architecture proposal after cut from original fMRI data for ERP classification.

Figure 14 displays the new architecture after the reduction, in which we compare the number of layers against the original architecture (Figure 13), where the removed dense layers are indicated in red. In this scenario, the reduction only occurred in one layer. This indicates that the input data involve a complex classification problem, where each layer is helping to solve the classification problem or the input features that are not suitable to solve the problem. After implementing the GCS architecture reduction, the values did not change much (Table 5). The number of hyperparameters decreased from 0.42 million to 0.41 million, and the testing error decreased from 23.05% to 21.03%. Thus, we could not conclude whether GCS helped to optimize the deep learning architecture. This may be due to different factors, such as the input data not having features that facilitate classification. The following subsection analyzes how TAM features with deep learning architectures can help us to solve this classification problem.

### 5.3. TAM Features and CNN Architecture Optimization by GCS for ERP Detection

**Figure 15:**
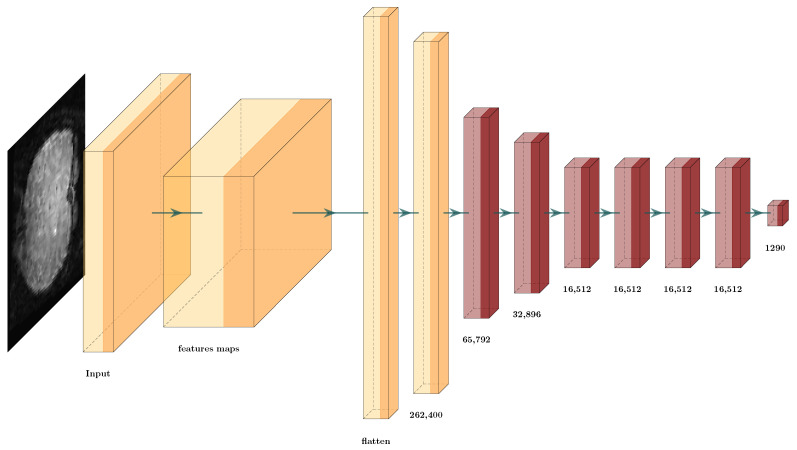
Architecture proposal using texture amortization map (TAM) features for ERP classification.

We tested deep learning architectures to classify ERPs. Thus, we evaluated the combination of deep learning architectures and the different TAM features to identify the best one to solve the ERP classification problem. We also used the GCS measure to evaluate the features obtained. For this, we processed each TAM feature separately, and introduced it into the proposed deep learning architecture. This created a scenario with which to test each of the different TAM features entropy, energy, contrast, and homogeneity with a deep learning architecture (Figure 15), and to use GCS architecture reduction. Table 7 shows the GCS scores from the data TAM layer features through all the layers.

**Figure 16:**
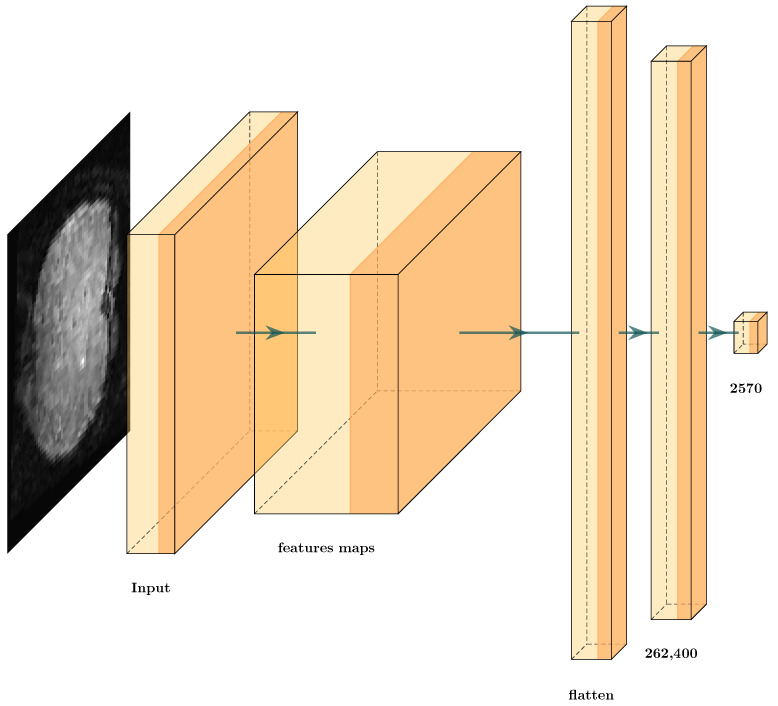
Architecture proposal using TAM features for ERP classification after cutting with the GCS measure.

Table 7 shows how the GCS measurements in each hidden layer slightly varied however, these variations are not incremental; they increase and decrease due to the different transformations through each layer. Additionally the GCS score shows that some layers obtained better features, and other transformations seem to add noise. Then, it was possible to reduce the proposed architecture to obtain Figure 16.

The results obtained by this reduction were compared to the original architecture that classified the TAM features individually in Table 8. We can observe that the value of the testing error did not vary substantially however, we observed that the same error values could be obtained with much smaller architectures (Figure 16). We compare the number of layers that we removed through GCS architecture reduction in red in Figure 15. Thus, the results confirm our original hypothesis. We evaluated the data transformations in the hidden layers and optimized the number of parameters with GCS architecture reduction. This provides opportunities to address countless questions and new ideas, for example, how to obtain the best optimization of this type of architecture. This is mentioned in the Discussion Section 6.

## 6. Discussion

The fMRI data obtained from brain ERP activity are not part of the standard data sets used in computer vision. These data change through time, so they are sensitive to the application of traditional filters. Thus, the use of the proposed TAM filter based on texture ideas improved the generation of new features due to the inherent adaptability of these filters. In this work, only some texture measures were analyzed because the main objective is the reduction of DNN architectures with the GCS and evaluating the impact that the TAM features can help on this reduction process. Thus, it is necessary to analyze the different measurements of textures that extract the best features within this case study for future work.

We proposed the novel GCS score to measure the ability of each layer in a deep architecture to classify MNIST and fMRI data sets. Even though additional tests need to be performed, we think that the GCS score can assist in the compression of simple feed-forward deep learning architectures. This is based on the GCS score, identifying which transformation/layer helps after the training process in the proposed architecture. This can be achieved as the GCS score helps to identify whether layers are learning noise or classifiable features, as demonstrated by the experiments, where the TAM and GCS score compressed the proposed deep architecture by at most 90%, while maintaining its accuracy performance.

We acknowledge that the proposed GCS score requires considerable improvement, in order to successfully identify the best architecture for the problem at hand, which takes a trained architecture and identifies which layers help to solve the problem. Nevertheless, this method can help to optimize simple feed-forward architectures, but further research is needed to integrate and improve the GCS score into the training process to identify not only layers, but also neurons helping in the classification process.

The detection of ERPs is a problem that has a future in neuroscientific research. In this work with TAM features, we demonstrated that they help to improve the accuracy of deep learning architectures. However, our future objective is to integrate the TAM features in the training of a CNN to focus the learned texture features on the ERP and lead the training stage with the GCS score. Here, the question is whether TAM features can optimize kernels within the convolution layer of a CNN to produce better features than TAMs.

For this, we propose:

1. To integrate the GCS score into the training process to neuron-level granularity;
2. To test the GCS score with different distances, other than Euclidean;
3. To integrate the GCS score in recurrent deep learning architectures; and
4. To integrate TAM features into the training stage of a CNN.

## Figures and Tables

**Table 1:** GCS values in layers 0 to 4 in CNN architectures in Figure 7 and Figure 9.

Layer Number	0	1	2	3	4
CNN (a)	conv	pool	conv	pool	conv
CNN-GCS (b)	0.0585	0.5465	0.6720	0.4655	0.9990
CNN (c)	conv	pool	conv	pool	flatten
CNN-GCS (d)	0.0005	0.0515	0.5460	0.7415	1.0

**Table 2:** GCS values in layers 5 to 9 in CNN architectures in Figure 7 and Figure 9.

Layer Number	5	6	7	8	9
CNN (a)	pool	flatten	fc	fc	fc
CNN-GCS (b)	0.4404	0.4404	0.9990	0.9999	1.0
CNN (c)	fc	fc	fc	fc	fc
CNN-GCS (d)	0.9884	0.9239	0.8820	0.9570	1.0

**Table 3:** GCS comparison of validation results between the original architecture and the cut architecture.

Architectures	Testing Error (%)	No. of Param.
CNN (a)	3.5	0.42 M
CNN-GCS (b)	2.8	0.06 M
CNN (c)	2.5	3.13 M
CNN-GCS (d)	2.4	0.05 M

**Table 4:** Percentage of error in image classification on MNIST.

Architecture	CNN + HFC	SOPCNN	VGG-5 (Spinal FC)	CNN GCS (b)	CNN GCS (d)
Testing Error (%)	0.16	0.17	0.28	2.8	2.4
No. of Params.	1.5 M	1.4 M	3.6 M	0.0 6M	0.05 M

**Table 5:** Parameter comparison of the architecture to detect ERPs.

Architecture	Parameters
Testing Error (%)	23.05
No. of Param.	0.42 M
Testing Error after cut (%)	21.03
No. of Param. after cut	0.41 M

**Table 6:** GCS measure for hidden layers in Figure 13.

layer 0	layer 1	layer 2	layer 3	layer 4	layer 5	layer 6	layer 7	layer 8
0.7516	0.8551	0.8389	0.8154	0.8465	0.8458	0.8598	0.9014	0.8802

**Table 7:** Measure of GCS in architecture for each texture feature.

layer	Entropy	Energy	Contrast	Homogeneity
0	0.8623	0.8830	0.8773	0.8342
1	0.9054	0.9124	0.9162	0.8932
2	0.9388	0.9722	0.9313	0.9260
3	0.9523	0.9733	0.9483	0.9413
4	0.9483	0.9225	0.9598	0.9414
5	0.9648	0.9563	0.9670	0.9541
6	0.9648	0.9563	0.9670	0.9541
7	0.9955	0.9877	0.9864	0.9873
8	0.9938	0.9879	0.9895	0.9911

**Table 8:** Architecture optimization based on feature maps.

Architecture	Error (%)	Error/Cut (%)	No. of Params.	No. of Params./Cut
Entropy	14.43	14.14	0.42 M	0.26 M
Energy	14.71	14.86	0.42 M	0.26 M
Contrast	17.59	15.14	0.42 M	0.26 M
Homogeneity	14.28	15.0	0.42 M	0.26 M

## Abbreviations

The following abbreviations are used in this manuscript:

CNN	Convolutional Neural Network
GCS	Geometric Classification Score
TAM	Texture Amortization Map
ERP	Event Related Potential
fMRI	Functional Magnetic Resonance Imaging
